# JNK-mediated microglial DICER degradation potentiates inflammatory responses to induce dopaminergic neuron loss

**DOI:** 10.1186/s12974-018-1218-1

**Published:** 2018-06-15

**Authors:** Qing Wang, Qian He, Yifei Chen, Wei Shao, Chao Yuan, Yizheng Wang

**Affiliations:** 1 Center of Cognition and Brain Science, Beijing Institute of Medical Sciences, Beijing, 100000 People’s Republic of China; 2 National Clinical Research Center for Aging and Medicine, Huashan Hospital, Fudan University, 12 Middle Wulumuqi Road, Shanghai, 200040 People’s Republic of China

**Keywords:** DICER, Microglia, Inflammation, Degradation, Phosphorylation

## Abstract

**Background:**

Amplified inflammation is important for the progression of Parkinson’s disease (PD). However, how this enhanced inflammation is regulated remains largely unknown. Deletion of DICER leads to progressive dopamine neuronal loss and induces gliosis. We hypothesized that the homeostasis of microglial DICER would be responsible for the amplified inflammation in the mouse model of PD.

**Methods:**

The microglia or C57BL/6 mice were treated or injected with l-methyl-4-phenyl-l,2,3,6-tetrahydropyridine (MPTP) or 1-methyl-4-phenylpyridinium (MPP^+^), respectively, for the model establishment. Microglia and astrocytes sorted by fluorescence-activated cell sorter (FACS) were assayed by quantitative real-time PCR, Western blotting, immunoprecipitation, enzyme-linked immunosorbent assay (ELISA), immunohistofluorescence, and mass spectrometry.

**Results:**

Microglial DICER was phosphorylated at serine 1456 by c-jun N-terminal kinase (JNK) and downregulated in response to 1-methyl-4-phenylpyridinium (MPP^+^), a causative agent in PD. Inhibition of JNK phosphorylation of DICER at serine 1456 rescued the MPP^+^-induced DICER degradation, suppressed microglial inflammatory process, and prevented the loss of tyrosine hydroxylase-expressing neurons in the mouse MPTP model.

**Conclusions:**

JNK-mediated microglial DICER degradation potentiates inflammation to induce dopaminergic neuronal loss. Thus, preventing microglial DICER degradation could be a novel strategy for controlling neuroinflammation in PD.

**Electronic supplementary material:**

The online version of this article (10.1186/s12974-018-1218-1) contains supplementary material, which is available to authorized users.

## Background

Parkinson’s disease (PD) is the second prevalent neurodegenerative disease worldwide and is characterized by the loss of dopaminergic neurons in the substantia nigra pars compacta (SNpc) [1]. Studies have shown that the development and progression of PD are accompanied by the microglial inflammatory process [2]. Activated microglia and elevated pro-inflammatory factors, such as tumor necrosis factor-alpha (TNF-α) and interleukin-1 beta (IL-1β), have been found in the brains of PD patients and various PD models [3–5]. Lipopolysaccharide (LPS), a bacterial endotoxin, can activate microglial inflammatory responses to induce degeneration of nigrostriatal dopamine neurons [6]. Anti-inflammatory agents, including naloxone and dexamethasone, can inhibit the activation of microglia and the production of pro-inflammatory cytokines to protect nigral dopaminergic neurons from degeneration [7, 8]. These observations suggest that manipulation of microglial inflammatory responses can alter disease progression. Therefore, exploration of the regulatory mechanism underlying the microglial inflammatory responses will provide important information to PD pathogenesis and treatment.

The 1-methyl-4-phenyl-1,2,3,6-tetrahydropyridine (MPTP) metabolite MPP^+^ is the causative agent in MPTP-induced parkinsonism in man and animals [9, 10]. MPTP can produce Parkinsonian-like syndrome in animal models [11]. Astrocytes convert MPTP into 1-methyl-4-phenylpyridinium (MPP^+^) which is uptaken by neurons to cause oxidative stress, and neuronal death and debris released from damaged neurons activate microglia to promote inflammation, leading to more neuronal loss [12]. Therefore, activated microglia has a crucial role in propagating this vicious cycle of neuronal damage. A recent work shows that MPP^+^ can potentiate TNF-α production in microglia by downregulating miR-7116-5p, a microRNA targeting TNF-α [9]. The important roles of microglial microRNAs in regulating inflammatory responses are also shown in various kinds of diseases, including experimental autoimmune encephalomyelitis, intracerebral hemorrhage, and amyotrophic lateral sclerosis [13–15]. These studies suggest that dysregulated microRNAs contribute to microglial inflammatory responses. As DICER, an RNase III family enzyme, is required for processing pre-miRNAs into mature microRNAs, exploring its potential involvement in microglial inflammatory responses might reveal novel insight into the molecular mechanism of inflammation in the PD brain.

Previous studies also show that artificial deletion of DICER in postmitotic dopamine neurons leads to progressive neuronal loss and that mice with DICER deletion in striatal neurons display ataxia, front and hind limb clasping, reduced brain size, and smaller neurons [16]. The DICER ablation in oligodendrocytes triggers gliosis and increases expression of inflammation markers [17]. The DICER deficit activates the NLRP3 inflammasome in the retinal pigmented epithelium [18]. However, whether DICER directly modulates microglial inflammatory responses in the MPTP model of PD remains unknown. In the current study, we report that MPP^+^ induces c-jun N-terminal kinase (JNK)-dependent and proteasome-mediated microglial DICER degradation to potentiate microglial inflammatory response and dopamine neuron loss. Our findings reveal a novel mechanism by which microglial inflammatory responses is controlled and potentiated in the PD models.

## Methods

### Animals

The C57BL/6 mice were from SLAK Laboratory Animal Shanghai, China. For the MPTP model, male wild-type C57BL/6 mice (8–10 weeks, *n* = 6 for each indicated group) were intraperitoneally injected with saline or l-methyl-4-phenyl-l,2,3,6-tetrahydropyridine (MPTP) at 20 mg/kg for four times with 2 h intervals in 1 day as reported previously [19]. The successful establishment of the model was confirmed as about 60% loss of dopaminergic (DA) neurons in SNpc [12]. For the peptide blocking experiments, the fusion peptides at 250 mg/kg TAT-scramble or TAT-DICER (by China Peptides) were co-injected with MPTP or saline into the mice. The mice were sacrificed for Western blotting analysis or immunohistofluorescence, 3 or 7 days, respectively, after MPTP or saline injection.

### Microglia and astrocytes sorting from adult mouse ventral midbrain

The ventral midbrain isolated from the brain of mice anesthetized was filtered through a 70-μm nylon cell strainer and digested with collagenase D (0.05%, Sigma, C5138) and DNase I (0.025 U/ml, Sigma) for 1 h at room temperature as reported previously [9]. After three washes with Hank’s Balanced Salt Solution (HBSS, Invitrogen) to remove the enzymes, cells were resuspended and then stained with allophycocyanin (APC)-labeled cluster of differentiation 45 (CD45)/Alexa Fluor 488-labeled crossing cluster of differentiation molecule 11B (CD11b), APC-labeled glutamate aspartate transporter (GLAST) antibodies, or Alexa Fluor 488/APC-labeled isotype at 4 °C for 1 h. CD11b^+^/ CD45^low^ or GLAST^+^ populations were then sorted by the flow cytometry (Cell Lab Quanta SC; Beckman Coulter, CA).

### Cell cultures, transfection, and drug treatment

The microglia and astrocytes were obtained from the cerebral cortices of C57BL/6 mouse pups postnatal day 0–1 as reported previously [9]. Briefly, the mouse cerebral cortices were isolated in cold phosphate-buffered saline (PBS), a water-based salt solution maintaining a constant pH, osmolarity, and ion concentrations and then digested for 6 min at 37 °C in 0.25% trypsin followed by adding culture medium to terminate digestion. After filtered through the 70 μm cell strainer, the suspensions were centrifuged at 1000 rpm for 3 min, and the pellets were then resuspended in Dulbecco’s modified Eagle’s medium (DMEM, Invitrogen) supplemented with 10% fetal bovine serum (FBS, Invitrogen) and antibiotics. Finally, the cells were cultured on 75 cm^2^ flasks. Seven to 9 days after seeding, the flasks were shaken at 200 rpm for 2–3 h. The cell suspension rich in microglia or the adhesive astrocytes was passed from the flasks into 12-well plates. The cells were used for experiments after 3–5 days. Primary cortical neurons were cultured from embryonic day 18 Sprague-Dawley rat brains and cultured in neurobasal supplemented with B27 and 0.5 mM Glutamax for 9–12 days as reported previously [20]. The BV2 cells and HEK293 cells were cultured in DMEM supplemented with 10% FBS and antibiotics (penicillin and streptomycin) in the 5% CO2 atmosphere at 37 °C.

All plasmids were transiently introduced into the cells using Lipofectamine 2000 (Invitrogen) following the manufacturer’s protocol.

All drugs added to cells or injected into the mice were made fresh, and the corresponding solvent was used as a control.

### RNA isolation and quantitative real-time PCR

The total RNA was extracted from microglia or BV2 cells using the Trizol reagent according to the manufacturer’s instructions. A total of 1 or 5 μg RNA from microglia or BV2 cells, respectively, were reverse-transcribed into cDNA using a reverse transcription system followed by quantitative real-time PCR (qRT-PCR) analysis. Target mRNA expression was quantified using the comparative Ct method (∆∆Ct method) and normalized to the reference gene level expression. The PCR primer sequences for mRNA analysis were as follows:

DICER: forward 5′-GGTCCTTTCTTTGGACTGCCA-3′, reverse 5′-GCGATGAACGTCTTCCCTGA-3′.

TNF-α: forward 5′-CTATGGCCCAGACCCTCACA-3′, reverse 5′-TTGAGATCCATGCCGTTGG-3′.

IL-1β: forward 5′-GCAACTGTTCCTGAACTCAACT-3′, reverse 5′-ATCTTTTGGGGTCCGTCAACT-3′.

COX-2: forward 5′-TGAGCAACTATTCCAAACCAGC-3′, reverse 5′-GCACGTAGTCTTCGATCACTATC-3′.

iNOS: forward 5′-GTTCTCAGCCCAACAATACAAGA-3′, reverse 5′-GTGGACGGGTCGATGTCAC-3′..

Actin: forward 5′-GGCTGTATTCCCCTCCATCG-3′, reverse 5′-CCAGTTGGTAACAATGCCATGT-3′.

The identities of the PCR products were confirmed by sequencing analysis.

### MTT assay

Microglia and BV2 cells were seeded on 12-well plates, and the medium was replaced with fresh DMEM or DMEM containing different concentration MPP^+^ for 12 h. The 3 (4,5-dimethylthiazol)-2-yl-2,5-diphenyl-tetrazolium bromide (MTT) solution (1 mg/ml MTT in phosphate-buffered saline) was added to each well for 4 h at 37 °C. Then, the solution was replaced by solvent dimethyl sulfoxide (DMSO) to solubilize the eventually formed formazan crystals. Optical absorbance was calculated by measuring optic density values at 570 nm.

### Western blotting

Total proteins extracted from cells were separated by SDS/7–10% PAGE gels and then transferred onto polyvinylidene difluoride (PVDF) membranes. After blocked with 5% non-fat milk in PBS at room temperature for 1 h, the membranes were incubated with the primary antibodies overnight at 4 °C. The primary antibodies were used at the following concentrations: DICER (1:1000), Ago 2 (1:5000), Drosha (1:500), ubquitin (1:1000), phosphorylated JNK (1:1000), JNK (1:1000), Caspase-3 (1:1000), cleaved Caspase-3 (1:500), ERK1/2 (1:1000), phosphorylated ERK (1:500), spectrin (1:2000), HA (1:2000), phosphorylated p38 (1:300), p38 (1:1000), and phospho Serine (1:200). The next day, the membranes were washed and then incubated with the secondary polyclonal antibodies conjugated with horseradish peroxidase at room temperature for 2 h. The protein bands were visualized with an ECL Western blotting substrate kit, and their densities were then quantified by densitometry analysis using ImageJ.

### Immunoprecipitation

Briefly, microglia or BV2 cells were harvested and extracted in the lysis buffer containing 2 mM EDTA or 20 mM Tris-HCl (pH 8.0), 137 mM NaCl, 10% glycerol, 1% NP40, and 2 mM EDTA. The antibody DICER (4 μg) was added into the lysates, and the mixtures were rotated at 4 °C overnight. Protein A/G beads were then added into the mixtures which were incubated for 2 h at room temperature. After washing three times with the lysis buffer, the immunoprecipitated proteins were analyzed using Western blotting.

### Enzyme-linked immunosorbent assay of TNF-α

The TNF-α levels in the medium of microglia after LPS and/or MPP^+^ were assessed by ELISA using a mouse TNF-α ELISA kit (BD Biosciences) according to the manufacturer’s instructions.

### Immunohistofluorescence and image analysis

The mice were perfused with saline followed by 4% paraformaldehyde (PFA) in 0.1 M phosphate buffer (PB) 7 days after saline or MPTP injection. The isolated brains were postfixed in 4% PFA and then dehydrated in 30% sucrose. Serial coronal sections were obtained at a thickness of 40 μM. Every fourth section of the entire slices was chosen to determine of the number of TH-positive neurons in SNpc and analysis of the fluorescence intensity of GFAP and Ibal 1. The sections were blocked with a normal donkey serum for 1 h and then were incubated with the primary antibodies (GFAP (1: 1000), Ibal 1 (1:1000), TH (1: 1000)) overnight at 4 °C. After addition of the secondary antibodies, the sections were incubated for 2 h at room temperature followed by three washes with PBS. Images were obtained using Nikon Ti microscope (Nikon, Japan). TH counts by Image Pro Plus was performed through unbiased stereology and the fluorescence intensity of GFAP and Iba1 quantified by ImageJ was defined as the average intensity of glial scar area in MPTP-injected mice or the SNpc area in saline-injected mice.

### DICER phosphorylation analysis by mass spectrometry

The phosphorylation of DICER was assayed by mass spectrometry as reported previously [21]. Briefly, immunoprecipitated samples from microglia and BV2 cells by the antibody against DICER or IgG were prepared. The precipitated proteins were separated by SDS/7% PAGE gels and then Coomassie-stained. The interesting bands migrating at a molecular weight of 220 kDa were cut for analysis. After destaining completely by destain solution (glacial acetic acid (10% (*v*/*v*)) and 30% (*v*/*v*) methanol), twice dehydrationby acetonitrile and the DICER bands were in-gel digested with trypsin overnight at 37 °C. After centrifugation, the supernatant-containing tryptic peptides were acidified with 5% formic acid and separated on a C18 column, and peptides were analyzed by Thermo Q Exactive HF Mass Spectrometer.

### Plasmid construction

The full-length sequences of mouse DICER from pCAGEN (Addgene, Plasmid #50558) were subcloned into the pCIG vector. A hemagglutinin (HA) epitope sequence (YPYDVPDYA) was fused to the amino terminus of DICER, and the plasmid sequences were confirmed by sequencing analysis.

### Antibodies and drugs

The following antibodies were used: TH (AB152) and spectrin (#Mab 1622) from Millipore; Iba1 (#01919741) from Wako; HA (H6908) and GFAP (G3893) from Sigma; Ago2 (sc-32877), Drosha (sc-33778), DICER (sc-30226), ubquitin (sc-8017), phosphorylated JNK (sc-6254), and JNK (sc-7345) from Santa Cruz Biotechnology; Caspase-3 (9662), cleaved Caspase-3 (#9664 L), ERK1/2 (9102), phosphorylated ERK (9101S), phosphorylated p38 (9216S), and p38 (8690S) from Cell Signaling Technology; phospho Serine (37430) from Qiagen; CD11b-Alexa Fluor 488 (557672) and CD45-APC (559864) from BD Biosciences; and GLAST-APC (130-098-803) from MiltenyiBiotec.

The following drugs were used: lipopolysaccharide (LPS, L2630), MPP^+^ (D048), MPTP (M0898), calpeptin (C8999) or MDL28170 (M6690), *N*-carbobenzoxyl-l-leucyl-l-leucyl-norvalinal (MG115, C6706), and lactacystin (L6785) from Sigma; U0126 (HY-12031), SP600125 (HY-12041), and SB203580 (HY-10256A) from Medchemexpress; and Z-DEVD-FMK (S7312) from Selleck.

### Statistics

Data were mean + SEM. All experiments were done independently at least three times. All statistical analyses were conducted using Office Excel 2007 and GraphPad Prism 5. Two-tailed Student’s *t* tests were used for comparisons between two groups and one-way ANOVA with Newman-Keuls post hoc test for more than two groups. Differences were considered to be significant when a *p* value is less than 0.05.

## Results

### Microglial DICER protein is specifically downregulated in the mouse MPTP model

**Fig. 1:**
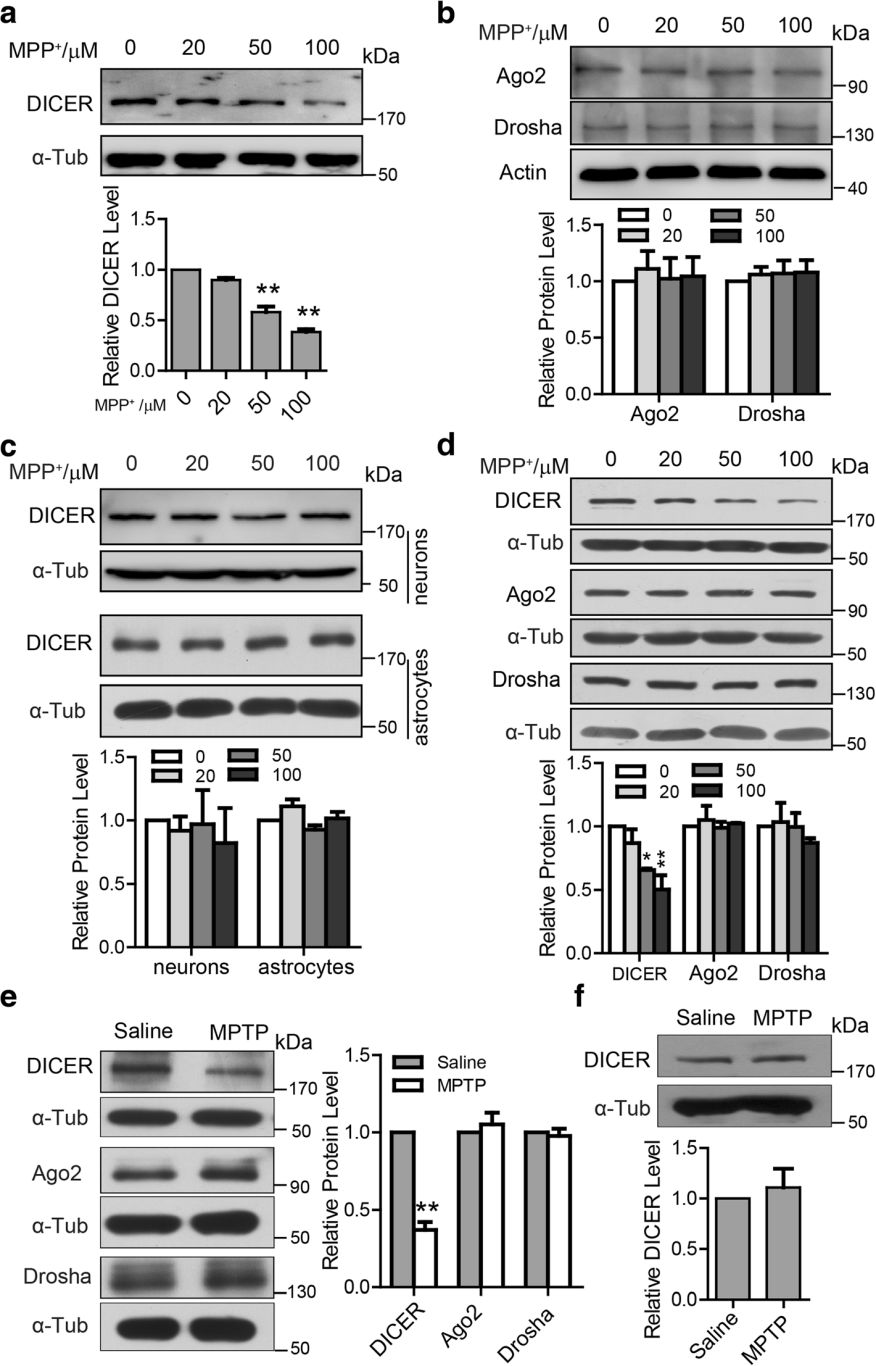
Microglial DICER protein is specifically downregulated in the MPTP model. **a**, **b** Upper—representative immunoblots of total lysates from the primary cultured microglia incubated with the indicated concentrations of MPP^+^ for 12 h and probed with the antibodies for DICER (**a**), Ago2, and Drosha (**b**). Lower—statistics (*n* = 4 for DICER, *n* = 5 for Ago2, and *n* = 4 for Drosha). **c** Upper—representative immunoblots of DICER from mouse neurons or astrocytes treated with the indicated concentrations of MPP^+^ for 12 h. Lower—statistics (*n* = 4 for neurons, *n* = 6 for astrocytes). **d** Upper—representative immunoblots of total lysates from the BV2 cells incubated with the indicated concentrations of MPP^+^ for 12 h and probed with the antibodies for DICER, Ago2, and Drosha. Lower—statistics (*n* = 5 for DICER and *n* = 4 for Ago2 and Drosha). **e** Left—representative immunoblots of total lysates from the CD11b+/CD45low-labeled microglia sorted from mouse ventral mesencephalon (VM) by flow cytometric 3 days after the administration of saline or MPTP and probed with the antibodies for DICER, Ago2, and Drosha. Right—statistics (*n* = 4 for DICER, Ago2, and Drosha). **f** Upper—representative immunoblots of total lysates from the GLAST+ labeled astrocytes sorted from VM by flow cytometric 3 days after administration of saline or MPTP and probed with the DICER antibody. Lower—statistics (*n* = 4 for DICER). Data are mean + SEM. *p < 0.05, **p < 0.01. Unless stated, α-tubulin (α-Tub) or Actin as loading controls. kDa, kilodalton

We first examined whether DICER expression is affected in microglia, neurons, or astrocytes in the mouse MPTP model. Exposure of the primary cultured microglia to MPP^+^ led to a great reduction in DICER protein levels (Fig. 1a). In contrast, the levels of other proteins important for microRNA processing, including Argonaute 2 (Ago2), the core component of RNA-induced silencing complex (RISC), and Drosha, a nuclear RNase III, remained unchanged in microglia treated with MPP^+^ (Fig. 1b). Further, DICER expression was not affected in the primary cultured mouse neurons or astrocytes in response to various concentrations of MPP^+^ (Fig. 1c). Moreover, there was no difference in microglia viability in the absence or presence of MPP^+^ for 12 h as assayed by the MTT test (Additional file 1a). These results provided the initial evidence to suggest that DICER expression is specifically suppressed in microglia by MPP^+^.

We then examined the effects of MPP^+^ on DICER expression in the BV2 cells, a murine microglial cell line that is commonly used for investigation of microglia function [22]. Similarly, decreased expression of DICER by MPP^+^ was evident, whereas that of Ago2 or Drosha was not observed (Fig. 1d). The MTT test showed that MPP^+^ did not affect the viability of BV2 cells (Additional file 1b). We then examined microglial DICER levels in the mouse MPTP model. We sorted microglia by fluorescence-activated cell sorting (FACS) and studied DICER expression in the microglia. We found that DICER expression was greatly suppressed in the cluster of differentiation molecule 11B (CD11b)-positive and CD45-low microglia in ventral mesencephalon (VM) of mice 3 days after MPTP injection. In contrast, the expression of Ago2 and Drosha was not changed (Fig. 1e). To clarify whether DICER expression in astrocytes is changed, we isolated glutamate aspartate transporter (GLAST, the astrocyte marker)-positive cells by FACS and found that there was no difference in DICER levels between saline- and MPTP-injected mice (Fig. 1f). Altogether, these results suggest that DICER proteins in microglia are specifically downregulated in the mouse MPTP model.

### Proteasome mediates microglial DICER downregulation induced by MPP^+^

**Fig. 2:**
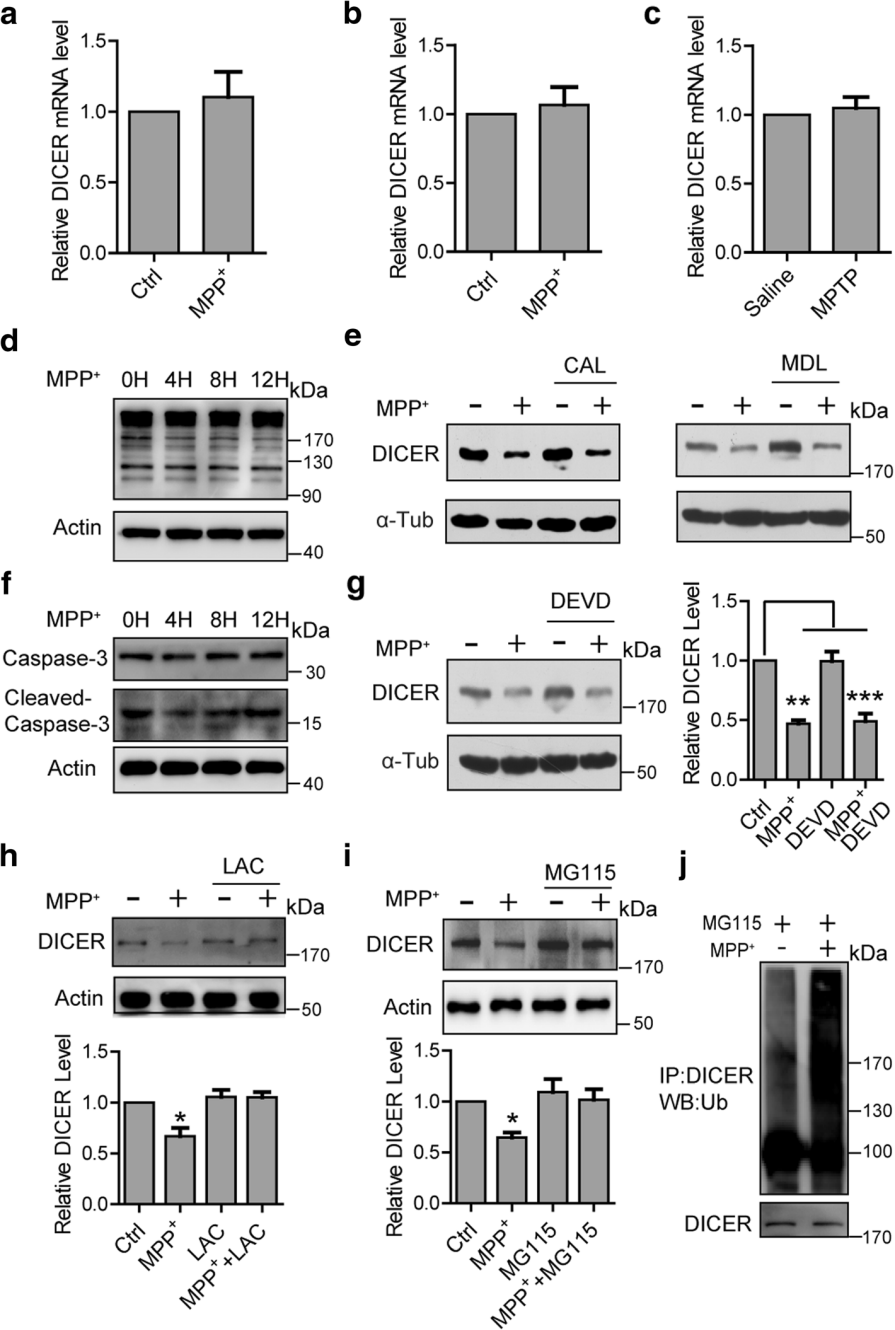
Inhibition of proteasome suppresses microglial DICER downregulation induced by MPP^+^. **a**, **b** Quantitative PCR analysis of DICER mRNA levels in microglia (**a**, *n* = 4) and BV2 cells (**b**, *n* = 4) treated with MPP^+^ (100 μM) for 12 h. **c** Quantitative PCR examination of DICER mRNA levels in microglia sorted from VM of mice after saline or MPTP injection (*n* = 4). **d** Representative immunoblots of total lysates from microglia treated with MPP^+^ for the indicated times with the antibody against α-spectrin. **e** Representative immunoblots of total lysates from BV2 cells incubated with MPP^+^ together with the calpeptin (CAL, 10 μM) or MDL28170 (MDL, 60 μM) for 12 h and probed with the indicated antibodies. **f** Representative immunoblots of total lysates from microglia treated with MPP^+^ for the indicated times using the antibodies against caspase-3 or cleaved caspase-3. **g** Left—representative immunoblots of total lysates from BV2 cells incubated MPP^+^ with/without Ac-DEVD-CHO (DEVD, 10 μM) and probed with the indicated antibodies. Right—statistics (*n* = 4). **h**, **i** Upper—representative immunoblots of total lysates from microglia treated with MPP^+^ with/without lactacystin (LAC, 1 μM, **h**) or *N*-carbobenzoxyl-l-leucyl-l-leucyl-norvalinal (MG115, 1 μM, **i**) for 12 h and probed with the indicated antibodies. Lower—statistics (*n* = 3 for LAC and *n* = 4 for MG115). **j** Representative immunoblots of immunoprecipitates of total lysates from microglia treated with/without MPP^+^ in the presence of MG115 and then probed with anti-ubiquitin antibody. Unless stated, 100 μM MPP^+^ was used. The corresponding mRNA levels were normalized to actin levels. Data are mean + SEM. *p < 0.05, **p < 0.01, ***p < 0.001

We next investigated whether the reduction in DICER protein level is due to its mRNA reduction. We measured DICER mRNA levels by quantitative real-time PCR (qRT-PCR) and observed that DICER mRNA levels were not changed in microglia (Fig. 2a) and BV2 cells (Fig. 2b) following MPP^+^ engagement. Similarly, DICER mRNA levels in FACS-isolated microglia from mice injected with MPTP were not changed compared with those from mice injected with saline (Fig. 2c). These results suggest that the reduction in DICER protein levels might be attributed to post-translational regulation. It has been reported that DICER can be degraded by proteases (calpain-1 and caspase-3) [23–25] or proteasome [26, 27]. We explored whether DICER reduction is due to either proteases or proteasome. As illustrated in Fig. 2d and Additional file 2a, the cleaved fragment of α-spectrin, one of the well-defined calpain substrates [28], was not observed in microglia or BV2 cells incubated with MPP^+^ for different times, suggesting that calpain is not activated, and hence, it is unlikely that calpain is involved in DICER reduction caused by MPP^+^. This notion was further supported by the findings that DICER reduction was not rescued by calpeptin or MDL28170, the calpain inhibitors (Fig. 2e and Additional file 2d), although they greatly inhibited glutamate-induced the cleavage of α-spectrin into the characteristic fragments in neurons (Additional file 2b). Additionally, caspase-3 activation was not observed in microglia incubated with MPP^+^ (Fig. 2f and Additional file 2c). Moreover, the addition of Z-DEVD-FMK (DEVD), an agent known to inhibit caspase-3, to the microglia did not affect MPP^+^-induced DICER reduction (Fig. 2g). However, MPP^+^-induced DICER reduction was markedly prevented when the microglia were incubated with lactacystin or *N*-carbobenzoxyl-l-leucyl-l-leucyl-norvalinal (MG115), agents known as proteasome inhibitors (Fig. 2h, i and Additional file 2e, f). These results suggest that proteasome participated in MPP^+^-induced DICER degradation. This assumption was further supported by the findings that DICER ubiquitination was greatly increased when microglia were incubated with MPP^+^ (Fig. 2j and Additional file 2g). Taken together, these observations suggest that MPP^+^-induced DICER reduction in microglia is due to its degradation mediated by proteasome.

### JNK is responsible for microglial DICER degradation induced by MPP^+^

**Fig. 3:**
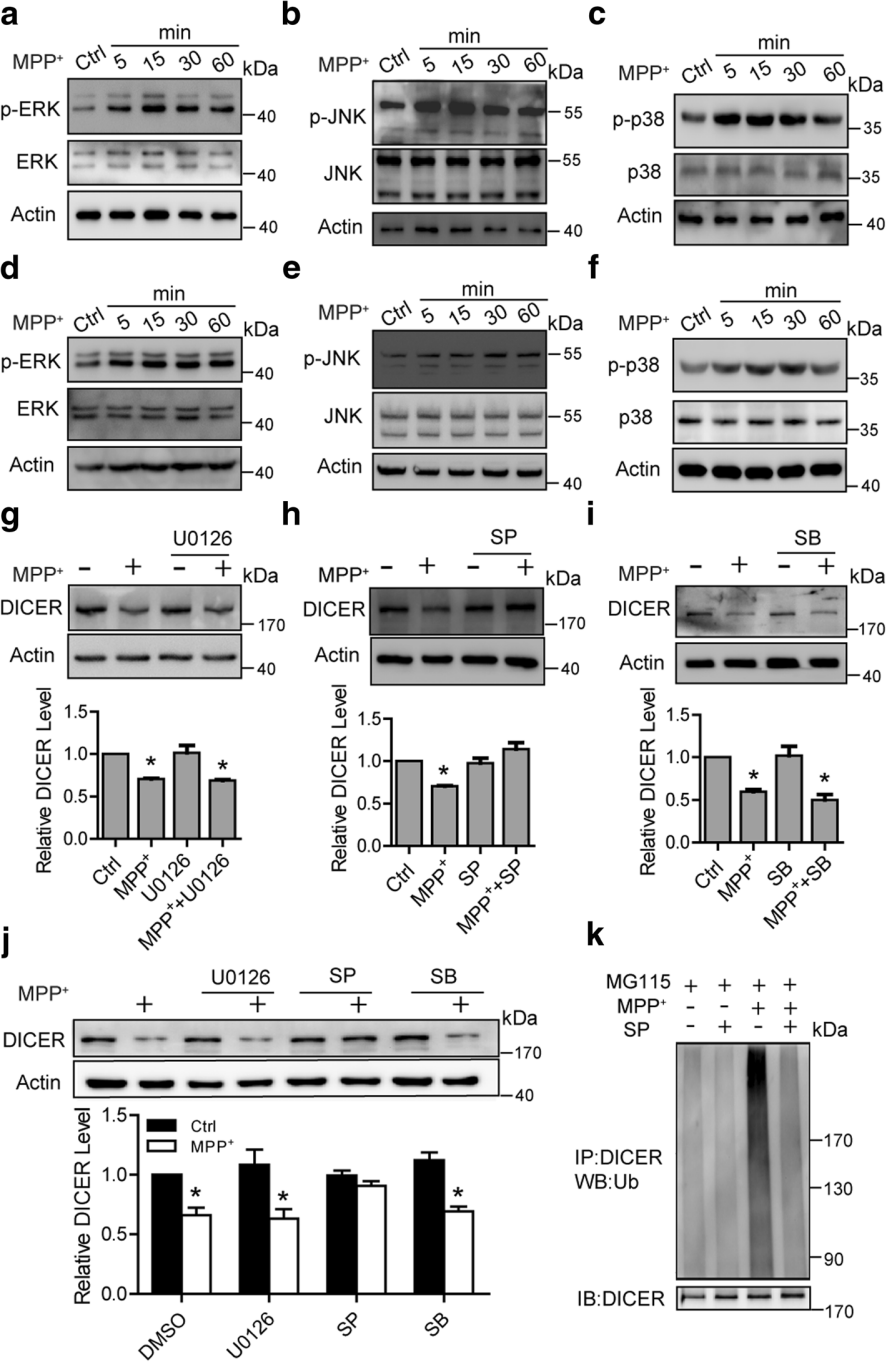
Inhibition of JNK suppresses microglial DICER degradation induced by MPP^+^. **a**–**f** Representative immunoblots of total lysates from microglia (**a**–**c**) and BV2 cells (**d**–**f**) exposed to MPP^+^ for the indicated times and probed with the antibodies for p-ERK and ERK (**a**, **d**), for p-JNK and JNK (**b**, **e**), and for p-p38 and p38 (**c**, **f**). **g**–**j** Representative immunoblots of total lysates from microglia (**g–i**) and BV2 cells (**j**) treated with MPP^+^ or/and U0126 (**g**, **j**,10 μM), SP600125 (SP, 10 μM) (**h**, **j**), and SB203580 (SB, 10 μM) (**i**, **j**) using the antibodies against DICER. Lower—statistics (**g**–**i**; *n* = 3 for **g**, *n* = 4 for **h**, and *n* = 3 for **i**, and *n* = 3 for **j**). **k** Upper—representative immunoblots of immunoprecipitates from total lysates of BV2 cells pretreated SP600125 for 2 h and then incubated with MG115 with/without MPP^+^ and immunoblotted with anti-ubiquitin antibody. Lower—immunoblots of the DICER precipitates. Data are mean + SEM. *p < 0.05

It has been reported that phosphorylation of a protein plays a role in its proteasomal degradation [29]. Treatment of microglia with MPP^+^ greatly increased the activities of mitogen-activated protein (MAP) kinases, including the extracellular signal-regulated kinases (ERKs, p42, and p44), the c-Jun N-terminal kinases (JNKs), and the p38 MAP kinases as evidenced by their respective phosphorylations. The ERK, JNK, and p38 MAPK were activated as early as 5 min after MPP^+^ engagement both in microglia (Fig. 3a–c) and BV2 cells (Fig. 3d–f). To assess the role of MAPKs in MPP^+^-induced DICER degradation, we then examined the effects of inhibition of these kinases on DICER degradation. Incubating microglia with U0126, SP600125, or SB203580, agents known as specific inhibitors to ERK, JNK, or p38 MAP kinase, respectively, markedly suppressed the phosphorylation of these kinases (Additional file 3a–c). The MPP^+^-induced DICER degradation was reversed when the microglia (Fig. 3h) or BV2 cells (Fig. 3j) were incubated with SP600125. In contrast, U0126 and SB203580 did not affect MPP^+^-induced DICER degradation (Fig. 3g–j). In addition, SP600125 eliminated MPP^+^-induced DICER ubiquitination (Fig. 3k). Together, these results provided the initial evidence to suggest that JNK might participate in MPP^+^-induced DICER degradation.

### Serine 1456 in microglial DICER is phosphorylated by JNK

**Fig. 4:**
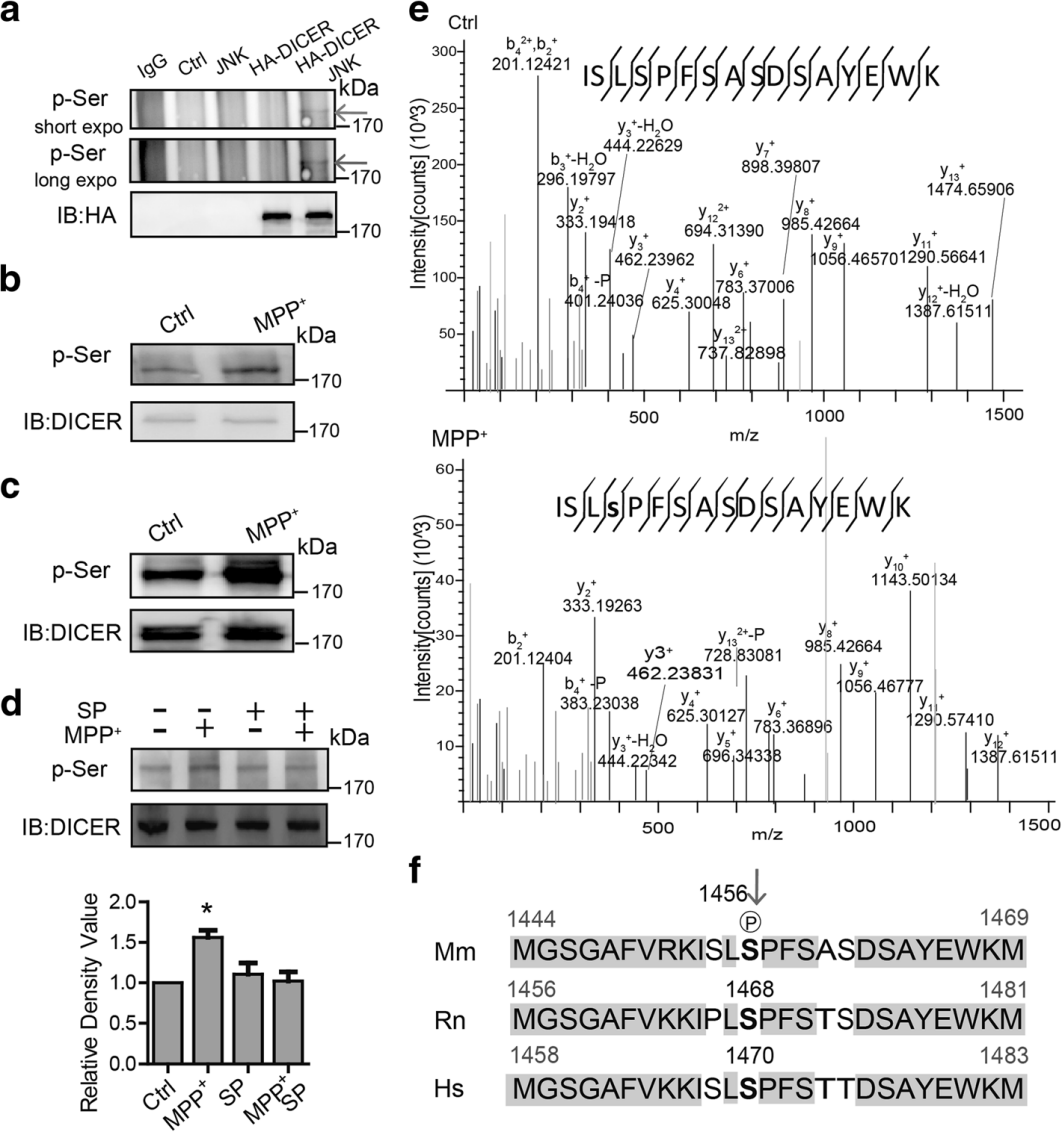
Microglial DICER is phosphorylated at serine 1456 by JNK. **a** Representative immunoblots of immunoprecipitates from the total lysates of HEK293 cells transfected with control (Ctrl), JNK, or/and HA-tagged DICER (HA-DICER) constructs and probed with anti-phospho Ser antibody. Lower—immunoblots of the precipitates with anti-HA antibody. **b**, **c** Representative immunoblots of immunoprecipitates of DICER from total lysates of microglia (**b**) and BV2 cells (**c**) exposed to MPP^+^ for 30 min and probed with the anti-phospho Ser antibody. **d** Upper—representative immunoblots of immunoprecipitates from total lysates of BV2 cells preincubated with SP600125 for 2 h then with MPP^+^ for 30 min and probed with the anti-phospho Ser antibody. Lower—statistics (*n* = 3). Data are shown as mean + SEM. *p < 0.05. **e** Mass spectrometry (MS) analysis of the DICER immunoprecipitated from the microglia incubated in the absence (upper, Ctrl) or presence (lower, MPP^+^) of MPP^+^ for 30 min. The phosphorylated serine residue was shown in lowercase bold letter. **f** Multiple sequence alignment of DICER containing the conserved serine residue in *Mus musculus* (Mm) (1444–1469), *Rattus norvegicus* (Rn) (1456–1481), and *Homo sapiens* (Hs) (1458–1483). Residues are colored based on percentage identity

We next examined whether JNK has a role in DICER phosphorylation and degradation. We transfected HEK293 cells with JNK and hemagglutinin (HA) epitope-tagged DICER expression constructs and immunoprecipitated whole cell lysates with HA antibody followed by immunoblotting using the antibody against phosphorylated serine. As shown in Fig. 4a, a phosphoserine signal at DICER position was detected, whereas no signal was observed without JNK transfection, suggesting that serine phosphorylation of DICER is JNK-dependent. Exposure of microglia to MPP^+^ caused an increase in phosphorylated serine signal migrating at DICER position (Fig. 4b). Similarly, elevation of serine phosphorylation of DICER was found in BV2 cells by MPP^+^ (Fig. 4c). Moreover, MPP^+^-induced serine phosphorylation of DICER was suppressed by SP600125 (Fig. 4d). Taken together, these results suggest that MPP^+^ activates JNK to phosphorylate DICER.

To clearly show that DICER is indeed phosphorylated by JNK, we used liquid chromatography-tandem mass spectrometry to further identify the phosphorylation site(s) of DICER in microglia exposed to MPP^+^. Mass spectrometry (MS) analysis of the DICER from MPP^+^-treated microglia (Fig. 4e) and BV2 cells (Additional file 4a) revealed that mouse DICER contained a phosphorylation site at serine 1456 (Ser 1456). This particular serine is highly conserved between human, mouse, and rat (Fig. 4f), suggesting that it is an important phosphorylation site for DICER. The bioinformatics prediction using Group-based Prediction software 3.0, a tool used to predict of kinase-specific phosphorylation sites [30], suggested that serine 1456 is a new candidate JNK phosphorylation site (Additional file 4b). Sequence analysis revealed that the Ser 1456 is followed by a proline, consistent with the notion that DICER is phosphorylated by JNK, a well-known proline-directed kinase. The MS analysis further revealed that MPP^+^ induced Ser 1456 phosphorylation, and the phosphorylation was abolished by SP600125 in microglia (Additional file 4c). Altogether, these results suggest that JNK indeed mediates DICER serine 1456 phosphorylation.

### Inhibiting DICER Ser 1456 phosphorylation prevents the MPP^+^-induced DICER degradation

**Fig. 5:**
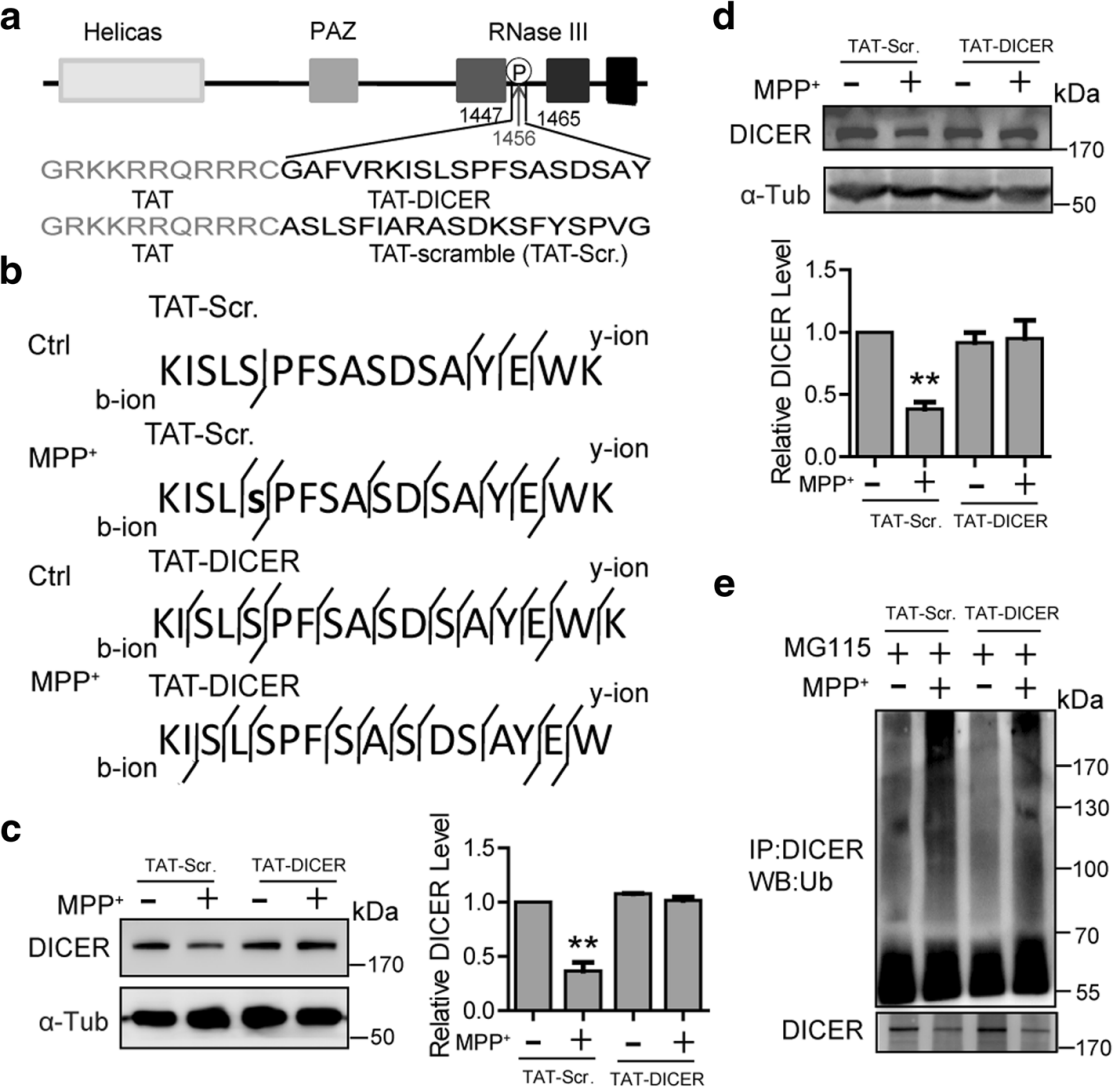
TAT-DICER prevents DICER degradation. **a** Schematic diagram of the TAT-DICER and TAT-scramble (TAT-Scr.) peptides. The letters with the light background indicate the 11 amino acid residues of the HIV transactivator protein (TAT). TAT was fused to the N terminus of the 19 amino acids containing Ser 1456 in mouse DICER. **b** MS analysis of the DICER immunoprecipitated from microglia pre-incubated with TAT-Scr. or TAT-DICER for 24 h and then with MPP^+^ for 30 min. The phosphorylated serine residue was shown in lowercase bold letter. (**c**, **d**) Upper—representative immunoblots of total cell lysates of microglia (**c**) or BV2 cells (**d**) incubated with TAT-Scr. or TAT-DICER for 24 h and then with MPP^+^ for 12 h. Lower—statistics (*n* = 4 for **c** and **d**). **e** Representative immunoblots of the immunoprecipitates of total cell lysates of BV2 cells incubated with TAT-Scr. or TAT-DICER for 24 h and then with MPP^+^ for another 8 h in the presence of MG115 and detected with anti-ubiquitin antibody. Data are mean + SEM. **p < 0.001

To provide clear evidence that Ser 1456 phosphorylation is indeed important for MPP^+^-induced DICER degradation, we designed a synthetic peptide comprising amino acid sequence 1448–1465 spanning the JNK phosphorylation site of mouse DICER, fused it to the protein transduction domain of the HIV-1 transactivator protein (TAT) and named it as TAT-DICER (Fig. 5a). Initial experiments were performed to investigate if the TAT-DICER could indeed affect Ser 1456 phosphorylation of DICER. As shown in Fig. 5b, TAT-DICER did inhibit Ser 1456 phosphorylation in microglia induced by MPP^+^. Moreover, the TAT-DICER also suppressed DICER degradation in microglia induced by MPP^+^ (Fig. 5c). Similar results were also obtained in BV2 cells (Fig. 5d). Consistently, TAT-DICER greatly inhibited MPP^+^-induced DICER ubiquitination (Fig. 5e). Collectively, these results suggest that JNK-mediated Ser 1456 phosphorylation plays a crucial role in DICER degradation.

### Microglial DICER degradation by MPP^+^ potentiates its inflammatory responses

**Fig. 6:**
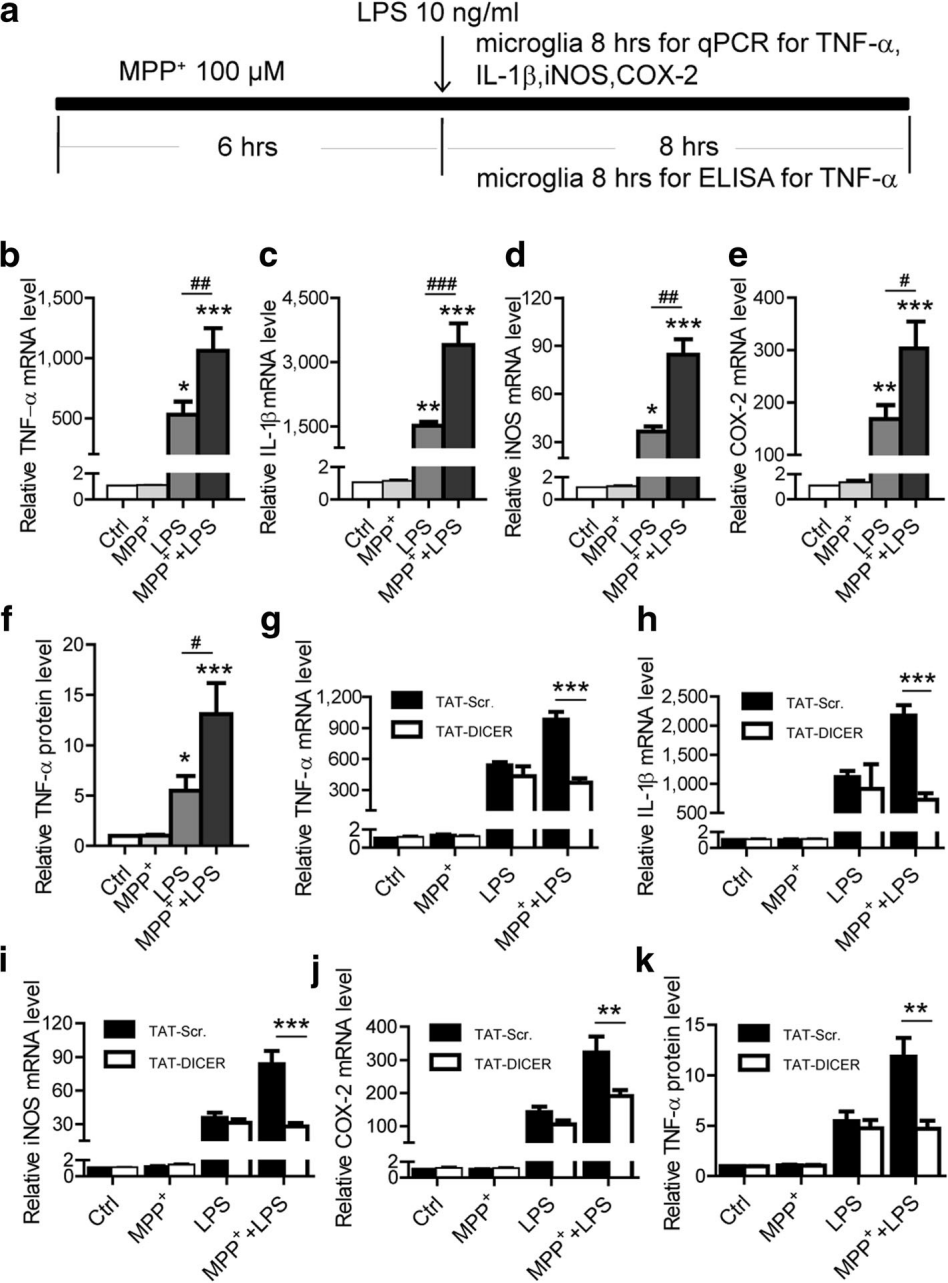
Degradation of DICER in microglia potentiates inflammatory responses. **a** The experimental diagram for **b**–**k**. Microglia were incubated with TAT-DICER or TAT- TAT-Scr. for 24 h and then with MPP^+^ and/or for the indicated time. **b**–**e** Quantitative PCR analysis of TNF-α (**b**, *n* = 4), IL-1β (**c**, *n* = 3), iNOS (**d**, *n* = 4), and COX-2 (**e**, *n* = 4) mRNA levels in microglia treated with MPP^+^ or/and LPS. **f** ELISA examination of protein levels of TNF-α in the supernatant of microglia treated with MPP^+^ or/and LPS. **g**–**j** Quantitative PCR analysis of TNF-α (**g**, *n* = 7), IL-1β (**h**), iNOS (**i**), and COX-2 (**j**, *n* = 8) mRNA levels in microglia incubated with TAT-Scr. or TAT-DICER for 24 h and then with MPP^+^ or/and LPS. **k** ELISA examination of TNF-α protein levels in the supernatant of microglia incubated with TAT-Scr. or TAT-DICER for 24 h then with MPP^+^ or/and LPS (*n* = 6). Data are mean + SEM. Comparisons between groups were performed with one-way ANOVA with Tukey’s post hoc test (**b**–**f**) or two-way ANOVA with Bonferroni post hoc test (**g**–**k**). *p < 0.05, **p < 0.01, ***p < 0.001. ^#^*p* < 0.05, ^##^*p* < 0.01, ^###^*p* < 0.001

Since microglia is the central immunocompetent cells in the brain [31], we investigated whether DICER degradation plays a role in microglial inflammatory responses. We evaluated the microglial activation by measuring the expression of pro-inflammatory mediators, including TNF-α,interleukin 1 beta (IL-1β), inducible nitric oxide synthase (iNOS) and cyclooxygenase-2 (COX-2) (Fig. 6a). The qRT-PCR analysis showed that mRNA levels of these factors were not affected by MPP^+^, suggesting that MPP^+^-regulated DICER degradation does not initiate the microglial inflammatory responses (Fig. 6b–e). Nevertheless, when microglial cells were incubated with lipopolysaccharide (LPS), an agent well known to activate microglia, the expression of these factors was increased (Fig. 6b–e). Moreover, the increased expression of these factors was greatly amplified when the microglia were pre-exposed to MPP^+^ (Fig. 6b–e). In addition, TNF-α protein level measured by enzyme-linked immunosorbent assay (ELISA) was also markedly potentiated (Fig. 6f). These results point to a possibility that DICER degradation caused by MPP^+^ leads to the hyper-microglial inflammatory responses.

To directly explore whether the reduction in DICER indeed has a role in microglial responses to LPS, we examined the effects of TAT-DICER on the production of these factors induced by LPS in the presence or absence of MPP^+^. The TAT-DICER did not affect the production of TNF-α, IL-1β, iNOS, and COX-2 induced by LPS (Fig. 6g–j). In contrast, the production of these factors potentiated by MPP^+^ was eliminated when microglia were incubated with TAT-DICER, but not with TAT-scramble (Fig. 6g–j). Additionally, exposure to TAT-DICER, but not to TAT-scramble, abolished MPP^+^-potentiated TNF-α protein production (Fig. 6k). Thus, DICER reduction induced by MPP^+^ greatly contributes to the amplification in microglial inflammatory responses.

### Inhibiting microglial DICER degradation limits its hyper-inflammatory responses and rescues TH-positive neuron loss in the mouse MPTP model

**Fig. 7:**
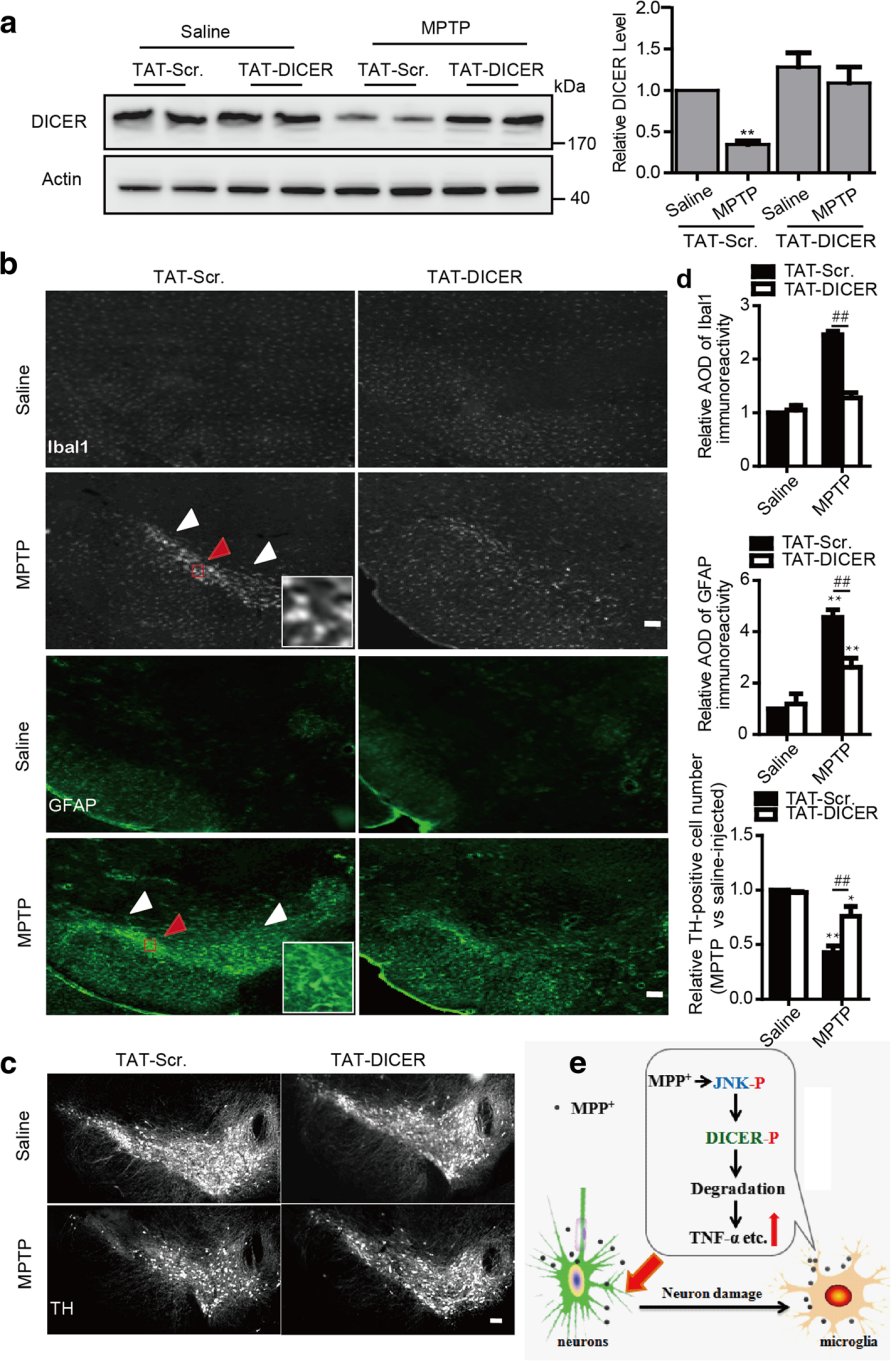
Inhibition of microglial DICER degradation suppresses inflammatory responses and reduces tyrosine hydroxylase-positive neuron loss in the mouse MPTP model. **a** Left—representative immunoblots of total lysates of microglia sorted from VM of mice 3 days after saline or MPTP injection together with TAT-Scr. or TAT-DICER and probed with the antibody against DICER. Right—statistics (*n* = 5). **b**, **c** Representative images of immunohistofluorescence staining with antibodies against Iba1 (top two, **b**) and GFAP (bottom two, **b**) in the ventral tegmental area and tyrosine hydroxylase (**c**) in the SNpc of mice 7 days after the administration of saline or MPTP with TAT-Scr. or TAT-DICER. In **b**, the images in the small squares magnified from the area indicated by the red arrows. Scale bar, 200 μM. AOD, average optical densities. **d** Statistics for **b** (top two, *n* = 6 for Ibal 1 and *n* = 5 for GFAP) and **c** (bottom, *n* = 4). **e** Schematic diagram of the working model. Microglial DICER phosphorylation and degradation by MPP^+^ amplified the inflammation caused by damaged neurons, leading to enhanced DA neuron loss. Data are mean + SEM. *p < 0.05, **p < 0.001, ^##^*p* < 0.01

The results above suggest that DICER in microglia is degraded in the MPTP models and that its degradation leads to the exaggerated inflammatory responses in cultured microglia. To show whether DICER degradation has a role in the regulation of microglial inflammatory responses in the mouse MPTP model, we injected TAT-DICER or TAT-scramble into the mice and examined their effects on DICER reduction and inflammation. Western blot analysis of the total lysates of the microglia isolated by FACS from the ventral mesencephalon revealed that the TAT-DICER remarkably inhibited DICER degradation in the mice injected with MPTP (Fig. 7a). Next, we detected microglial or astrocytic activation as evidenced by ionized calcium-binding adapter molecule 1 (Ibal1) or glial fibrillary acidic protein (GFAP) immunohistofluorescence staining, respectively. Neither TAT-scramble nor TAT-DICER itself affected Ibal1 and GFAP expression. In contrast, the expression of Ibal1 and GFAP was increased in mice injected with MPTP, and the increased expression of Ibal1 and GFAP was markedly inhibited by TAT-DICER, but not by TAT-scramble (Fig. 7b, d).

To determine whether TAT-DICER-inhibited glial activation has a role in nigral dopaminergic neuron loss in the mouse MPTP model, we counted tyrosine hydroxylase (TH, a marker for dopamine neurons)-positive neuron number in the mice. The numbers of TH-positive neurons were greatly reduced in the mouse MPTP model and TAT-DICER markedly prevented the reduction in the number of TH-positive neurons caused by MPTP (Fig. 7c, d). Taken together, these results suggest that microglial DICER degradation in the mouse MPTP model greatly contributes to glial hyperactivation and TH-positive neuron loss.

## Discussion

In this study, we report JNK-mediated degradation of DICER by neurotoxin MPP^+^ and its crucial role in microglial hyper-inflammatory responses to cause dopamine (DA) neuron loss. Several lines of experimental results support this conclusion. First, microglial DICER was specifically degraded via the proteasomal pathway in response to MPP^+^. Second, MPP^+^ caused JNK activation and inhibition of JNK activities stopped microglial DICER degradation. Third, JNK phosphorylated DICER at serine 1456. Fourth, inhibition of microglial DICER Ser 1456 phosphorylation prevented its degradation as well as its hyper-inflammatory responses. Last, inhibition of microglial DICER degradation limited its hyperactivation and reduced DA neuron loss in the mouse MPTP model. Since specific and progressive dopaminergic neuron loss is one of the key characteristics of the PD pathology, the microglial DICER degradation suggests a particular role of this specific regulation of DICER in the pathogenesis of PD. Thus, preventing microglial DICER degradation may be a new strategy to curb microglial hyper-inflammatory responses and DA neuron loss in PD (Fig. 7e).

The DICER has important roles in diverse biological processes, including chromatin structure remodeling, immune responses, and stem-cell maintenance and differentiation [32–35]. Therefore, DICER expression level should be tightly controlled. At the post-translational level, DICER is glycosylated to maintain its intracellular level and proper folding [36]. Smoking-induced DICER SUMOylation in lung macrophages results in abnormal profiles of microRNA expression [37]. Phosphorylation of DICER on Ser 1705 and Ser 1833 by ERK triggers its nuclear localization to coordinate oocyte-to-embryo transition in the development of *Caenorhabditis elegans* [38]. Here, we showed that JNK phosphorylation of microglial DICER at Ser 1456 induced by neurotoxin MPP^+^ promotes its degradation. Injection of TAT-DICER, a cell-permeable peptide with the ability to pass the blood-brain barrier [39–41], prevented DICER phosphorylation and degradation. Thus, DICER is phosphorylated by different kinases to function in different biological processes. It should be pointed out that although JNK activation is found in neurons, astrocytes [42, 43], and microglia, DICER degradation induced by MPP^+^ was observed only in microglia. It is thus possible that an E3 ligase specific for DICER is present only in microglia. This assumption is consistent with the findings that inhibition of DICER phosphorylation by TAT-DICER suppressed its ubiquitination and degradation in microglia induced by MPP^+^.

Previous studies reported that a genetic deletion of DICER in forebrain excitatory neurons or oligodendrocytes leads to astrogliosis and microgliosis [17, 44], while little is known about the role of DICER in microglia. One important finding of the current study is that microglial DICER degradation potentiated its inflammatory responses. A recent study showed that DICER knock-out in adult microglia leads to hyperactivation of these cells after LPS challenges [45]. Therefore, it is likely that DICER can affect the inflammation in cell autonomous and non-cell autonomous manners. In this context, inhibiting DICER reduction to curb inflammation is a novel approach to specifically affect the inflammatory process. It remains unclear how DICER affects microglial inflammatory responses. Our recent work reports that miR-7116-5p, a microRNA targeting TNF-α, is downregulated by MPP^+^ and potentiates TNF-α production in microglia [9]. Therefore, changes in microRNA expression likely play an important role in DICER deficit-induced inflammation. Additionally, DICER deficit in the retinal pigmented epithelium induces accumulation of Alu RNA to activate NLRP3 inflammasome [18, 46]. It is not clear whether microglial hyperactivation responses induced by DICER degradation are due to the long non-coding RNA Alu-dependent mechanism.

From the lessons of the current clinical trials of Alzheimer’s disease [47], it is believed that there is no beneficial effect to inhibit inflammation in late-stage patients who have been with obvious symptoms, and it is therefore necessary to select a right time to curb the amplified inflammation. A key finding of the current study is that DICER is degraded at the early time in the pathogenic process in the mouse MPTP model. Consistently, preventing DICER degradation at this stage significantly suppressed glial activation and improved DA neuron survival. Additionally, pre-treating mice with eicosanoyl-5-hydroxytryptamide, which had a direct anti-inflammatory effect, for 4 weeks showed a robust protection for dopaminergic neurons following MPTP challenge [48], and co-administration anti-inflammatory drug MDG548 injected 15 min before MPTP prevented DA neuron loss [49]. Moreover, the use of ibuprofen, a compound known to inhibit inflammation, is associated with lowering PD risk [50]. Therefore, the inhibition of inflammation by the early inhibition of DICER degradation would be a promising strategy to prevent neurodegeneration.

## Conclusions

Microglial DICER is phosphorylated by JNK to induce its degradation, potentiate microglial inflammatory processes, and enhance DA neuron damage. These observations revealed an unknown regulatory mechanism of microglial DICER and its role in promoting microglial hyperactivation of inflammation in the mouse MPTP model. The demonstration of JNK-mediated DICER degradation in the brain will further expand our understanding of the amplification of microglial inflammation.

## Additional files

Additional file 1: Related to Fig. 1 (a and b) The viability of microglia (a, *n* = 3) and BV2 cells (b, *n* = 3) determined by MTT assay 12 h after MPP^+^ treatment. Data shown as mean + SEM. (PNG 17 kb)

Additional file 2: Related to Fig. 2 (a) Representative immunoblots of total lysates of BV2 cells treated with 100 μM MPP^+^ for the indicated times with the antibody against α-spectrin. (b) Representative immunoblots of total lysates of primary cultured neurons treated with 50 μM glutamate (Glu) or/and calpeptin (CAL) or MDL28170 (MDL) for 4 h with the antibody against α-spectrin. (c) Representative immunoblots of total lysates of BV2 cells treated with MPP^+^ for the indicated times with the antibody against caspase-3 and cleaved caspase-3. (d) Statistics of DICER in Fig. 2e. (e) Upper, representative immunoblots of total lysates from BV2 cells treated with 100 μM MPP^+^ with/without LAC (1 μM) (e) or MG115 (1 μM) (f) for 12 h and detected with the indicated antibodies. Lower: statistics. (g) Representative immunoblots of immunoprecipitates for total lysates of BV2 cells treated with MG115 with or without MPP^+^ and then probed with anti-ubiquitin antibody. Data are shown as mean + SEM. *p < 0.05, **p < 0.01, ***p < 0.001. α-tubulin (α-Tub), a loading control. kDa, kilodalton. (PNG 512 kb)

Additional file 3: Related to Fig. 3 (a–c) Representative immunoblots of total lysates of BV2 cells treated with U0126 (a), SP (b), and SB (c) with indicated antibodies. α-tubulin (α-Tub), a loading control. kDa, kilodalton. (PNG 54 kb)

Additional file 4: Related to Fig. 4 (a) Mass spectrometry (MS) analysis of the DICER immunoprecipitated from BV2 cells treated with MPP^+^ for 30 min. The phosphorylated serine residue as revealed by MS is shown in lowercase bold letter. (b) Table representing a list of JNK phosphorylation sites of DICER predict by GPS3.0. (c) MS analysis of the DICER immunoprecipitated from microglia pre-incubated with or without SP for 2 h and then with MPP^+^ for 30 min. The phosphorylated serine residue as revealed by MS is shown in lowercase bold letter. (PNG 289 kb)

## Abbreviations

Ago2: Argonaute 2
APC: Allophycocyanin
CD11b: Crossing cluster of differentiation molecule 11B
CD45: Cluster of differentiation 45
COX-2: Cyclooxygenase-2
DEVD: Z-DEVD-FMK
ELISA: Enzyme-linked immunosorbent assay
ERK: Extracellular signal-regulated kinase
FACS: Fluorescence-activated cell sorter
FBS: Fetal bovine serum
GFAP: Glial fibrillary acidic protein
GLAST: Glutamate aspartate transporter
Ibal1: Ionized calcium-binding adapter molecule 1
IL-1β: Interleukin-1 beta
iNOS: Inducible nitric oxide synthase
JNK: c-jun N-terminal kinase
LPS: Lipopolysaccharide
MAP: Mitogen-activated protein
MG115: *N*-carbobenzoxyl-l-leucyl-l-leucyl-norvalinal
MPP^+^: 1-Methyl-4-phenylpyridinium
MPTP: l-Methyl-4-phenyl-l,2,3,6-tetrahydropyridine
MS: Mass spectrometry
MTT: 3 4,5-Dimethylthiazol-2-yl-2,5-diphenyl-tetrazolium bromide
PBS: Phosphate-buffered saline
PD: Parkinson’s disease
PFA: Paraformaldehyde
PVDF: Polyvinylidene difluoride
qRT-PCR: Quantitative real-time PCR
SNc: Substantia nigra compacta
TH: Tyrosine hydroxylase
TNF-α: Tumor necrosis factor-alpha
VM: Ventral mesencephalon
