# Supplementary figures and images for: JNK-mediated microglial DICER degradation potentiates inflammatory responses to induce dopaminergic neuron loss
Source: J Neuroinflammation. 2018 Jun 15;15:184. doi: 10.1186/s12974-018-1218-1 (PMC6003208; doi:10.1186/s12974-018-1218-1)

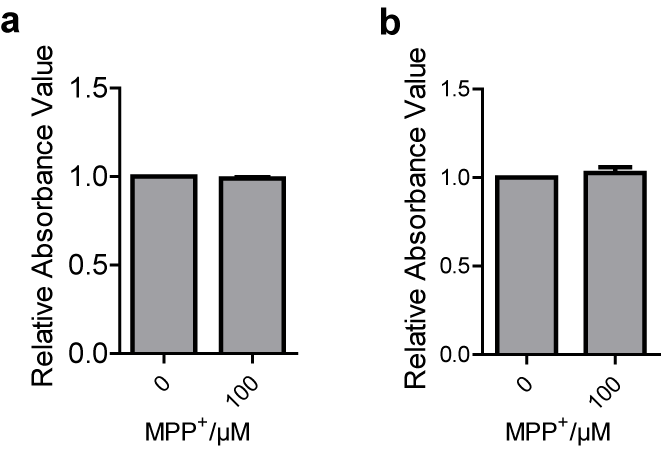

Supplement: Supplementary file 1 — Related to Fig. 1 (a and b) The viability of microglia (a, n = 3) and BV2 cells (b, n = 3) determined by MTT assay 12 h after MPP+ treatment. Data shown as mean + SEM. (PNG 17 kb) [file 12974_2018_1218_MOESM1_ESM.png]

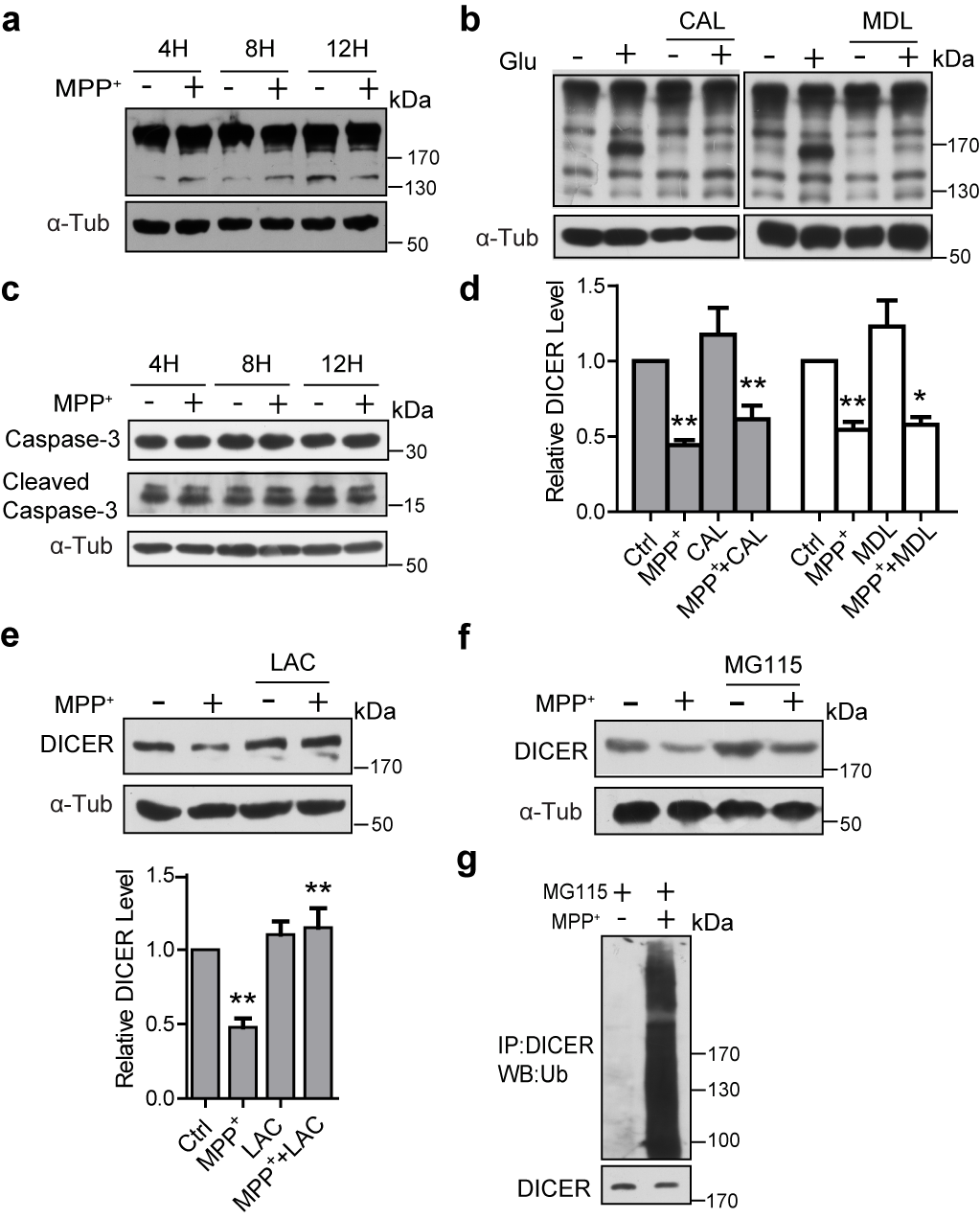

Supplement: Supplementary file 2 — Related to Fig. 2 (a) Representative immunoblots of total lysates of BV2 cells treated with 100 μM MPP+ for the indicated times with the antibody against α-spectrin. (b) Representative immunoblots of total lysates of primary cultured neurons treated with 50 μM glutamate (Glu) or/and calpeptin (CAL) or MDL28170 (MDL) for 4 h with the antibody against α-spectrin. (c) Representative immunoblots of total lysates of BV2 cells treated with MPP+ for the indicated times with the antibody against caspase-3 and cleaved caspase-3. (d) Statistics of DICER in Fig. 2e. (e) Upper, representative immunoblots of total lysates from BV2 cells treated with 100 μM MPP+ with/without LAC (1 μM) (e) or MG115 (1 μM) (f) for 12 h and detected with the indicated antibodies. Lower: statistics. (g) Representative immunoblots of immunoprecipitates for total lysates of BV2 cells treated with MG115 with or without MPP+ and then probed with anti-ubiquitin antibody. Data are shown as mean + SEM. *p < 0.05, **p < 0.01, ***p < 0.001. α-tubulin (α-Tub), a loading control. kDa, kilodalton. (PNG 512 kb) [file 12974_2018_1218_MOESM2_ESM.png]

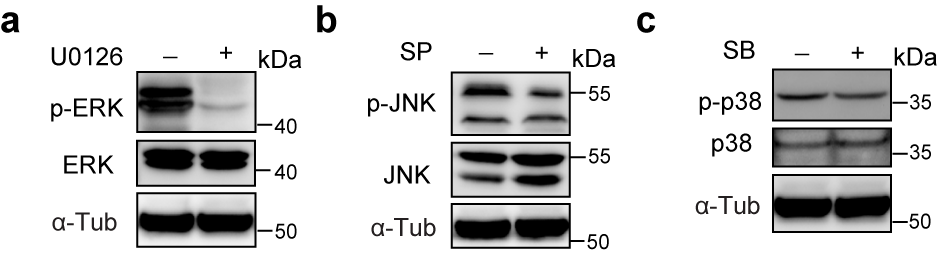

Supplement: Supplementary file 3 — Related to Fig. 3 (a–c) Representative immunoblots of total lysates of BV2 cells treated with U0126 (a), SP (b), and SB (c) with indicated antibodies. α-tubulin (α-Tub), a loading control. kDa, kilodalton. (PNG 54 kb) [file 12974_2018_1218_MOESM3_ESM.png]

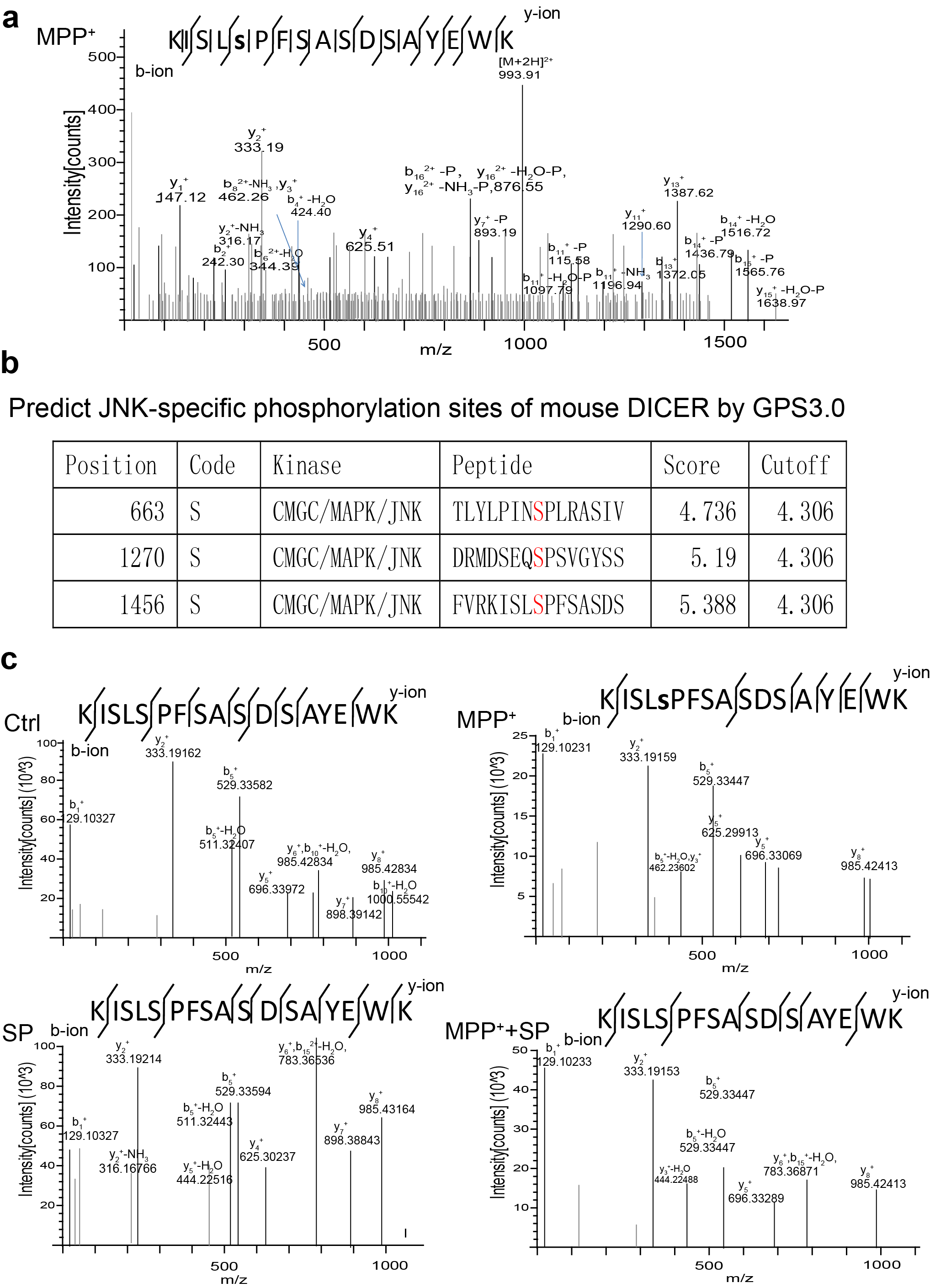

Supplement: Supplementary file 4 — Related to Fig. 4 (a) Mass spectrometry (MS) analysis of the DICER immunoprecipitated from BV2 cells treated with MPP+ for 30 min. The phosphorylated serine residue as revealed by MS is shown in lowercase bold letter. (b) Table representing a list of JNK phosphorylation sites of DICER predict by GPS3.0. (c) MS analysis of the DICER immunoprecipitated from microglia pre-incubated with or without SP for 2 h and then with MPP+ for 30 min. The phosphorylated serine residue as revealed by MS is shown in lowercase bold letter. (PNG 289 kb) [file 12974_2018_1218_MOESM4_ESM.png]
